# Risk factors for the progression of trachomatous scarring in a cohort of women in a trachoma low endemic district in Tanzania

**DOI:** 10.1371/journal.pntd.0009914

**Published:** 2021-11-19

**Authors:** Meraf A. Wolle, Beatriz E. Muñoz, Fahd Naufal, Michael Saheb Kashaf, Harran Mkocha, Sheila K. West

**Affiliations:** 1 Dana Center for Preventive Ophthalmology, Wilmer Eye Institute, Johns Hopkins University, Baltimore, Maryland, United States of America; 2 Kongwa Trachoma Project, Kongwa, United Republic of Tanzania; RTI International, UNITED REPUBLIC OF TANZANIA

## Abstract

**Background:**

Trachoma, a chronic conjunctivitis caused by *Chlamydia trachomatis*, is the leading infectious cause of blindness worldwide. Trachoma has been targeted for elimination as a public health problem which includes reducing trachomatous inflammation—follicular prevalence in children and reducing trachomatous trichiasis prevalence in adults. The rate of development of trachomatous trichiasis, the potentially blinding late-stage trachoma sequelae, depends on the rate of trachomatous scarring development and progression. Few studies to date have evaluated the progression of trachomatous scarring in communities that have recently transitioned to a low trachomatous inflammation—follicular prevalence.

**Methodology/Principal findings:**

Women aged 15 and older were randomly selected from households in 48 communities within Kongwa district, Tanzania and followed over 3.5 years for this longitudinal study. Trachomatous inflammation—follicular prevalence was 5% at baseline and at follow-up in children aged 1–9 in Kongwa, Tanzania. 1018 women aged 15 and older had trachomatous scarring at baseline and were at risk for trachomatous scarring progression; 691 (68%) completed follow-up assessments. Photographs of the upper tarsal conjunctiva were obtained at baseline and follow-up and graded for trachomatous scarring using a previously published four-step severity scale. The overall cumulative 3.5-year progression rate of scarring was 35.3% (95% CI 31.6–39.1). The odds of TS progression increased with an increase in age in women younger than 50, (OR 1.03, 95% CI 1.01–1.05, p = 0.005) as well as an increase in the household poverty index (OR 1.29, 95% CI 1.13–1.48, p = 0.0002).

**Conclusions/Significance:**

The 3.5-year progression of scarring among women in Kongwa, a formerly hyperendemic now turned hypoendemic district in central Tanzania, was high despite a low active trachoma prevalence. This suggests that the drivers of scarring progression are likely not related to on-going trachoma transmission in this district.

## Author summary

Trachoma, a chronic conjunctivitis caused by *Chlamydia trachomatis*, presents with follicles (trachomatous inflammation—follicular, TF) in children which leads to trachomatous conjunctival scarring (TS) in young adults. TS can progress to the in-turning of eyelashes, trachomatous trichiasis (TT) which places individuals at high risk of irreversible vision loss. Few studies to date have evaluated the progression of TS in communities that have recently transitioned to a low trachoma prevalence.

We studied the progression of TS in women in Kongwa, Tanzania a district that recently transitioned to a low prevalence of trachoma. We found that the overall cumulative progression of scarring was 35.3% over 3.5 years. The scarring progression rate observed is very similar to what we observed a decade prior in Kongwa when the trachoma prevalence was very high.

Our findings suggest that once scarring has developed it continues to progress irrespective of the current trachoma environment. This has potential ramifications for trachoma elimination efforts. An area could achieve the elimination of TF and still have to deal with scarring progression, which may lead to the development of TT. If this occurs: 1) elimination of TT will be delayed which will delay the overall elimination of trachoma as a public health problem, and 2) the limited resources available to elimination programs may need to be re-allocated.

## Introduction

Trachoma, a chronic conjunctivitis caused by *Chlamydia trachomatis (C*. *trachomatis)*, is the leading infectious cause of blindness worldwide [1]. 142 million people are at risk of blindness, and 1.9 million adults are visually impaired or irreversibly blind, from trachoma [1]. *C*. *trachomatis* conjunctivitis presents with follicles (trachomatous inflammation—follicular, TF) and inflammatory thickening of the conjunctiva (trachomatous inflammation—intense, TI) in the acute, active, stage of infection [2]. Repeat bouts of active trachoma in children lead to trachomatous conjunctival scarring (TS) in young adults. TS can progress further to the in-turning of the eyelid, entropion, and the in-turning of eyelashes, trachomatous trichiasis (TT). If left uncorrected, TT places individuals at high risk of irreversible vision loss from corneal opacification (CO) [1,3,4]. Both TT and CO, the late stages of trachoma, are more prevalent in women. Women are up to four times more likely than men to develop more severe trachoma sequelae that require surgical correction and are more likely to be disabled from end-stage trachomatous disease [5–7].

The World Health Organization (WHO) has targeted trachoma for elimination as a public health problem which includes reducing TF rates in children as well as reducing TT prevalence in adults [8,9]. To eliminate trachoma, the WHO has advocated using the SAFE strategy: ‘**S**urgery’ for TT, ‘**A**ntibiotics’ to clear infection, ‘**F**acial cleanliness’ to reduce transmission, and ‘**E**nvironmental improvement’ specifically improving access to water and sanitation [3]. With the SAFE strategy, some trachoma elimination programs have successfully met the trachoma elimination targets; however, 44 countries still remain trachoma endemic [1].

Part of the difficulty in eliminating trachoma is that the extent to which the rate of trachoma disease progression to TT is affected by declining TF and *C*. *trachomatis* infection remains unknown. The rate of development of TT, the potentially blinding late-stage trachoma sequelae, depends on the rate of TS development and progression. Thus, determining how TS development and progression is affected by declining TF rates will inform how the late stage sequalae, including TT, may be affected in areas where people have been exposed to higher rates of *C*. *trachomatis* infection and TF as children but now live in a trachoma hypoendemic area.

Longitudinal studies have shown that TS can progress without evidence of either continued individual re-infection with *C*. *trachomatis* or individual signs of active trachoma (TF) [10,11]. Our previous longitudinal study in Tanzania showed that incident TS can develop in women who live in communities with low active trachoma [12]. However, few studies to date have evaluated the progression of existing TS in communities that have recently transitioned to a low TF prevalence.

The goal of this study is to determine the progression of existing TS over 3.5 years in a cohort of women living in Kongwa, a formerly trachoma hyperendemic district, where the TF prevalence of the district was 5%.

## Methods

### Ethics statement

All study procedures were reviewed and approved by the Johns Hopkins Institutional Review Board and the Tanzanian National Institute for Medical Research. Community leaders in each village provided verbal consent permitting community participation in the study. Written informed individual consent was then obtained from all participants. For those few participants between age 15 and age 18, parental consent was not obtained as it was not required since the women were married and no longer living at their parental homes. English consent forms were translated into Swahili and then back translated into English. Study personnel solicited informed consent in Swahili, or in the local languages if necessary. This study fully complied with the precepts of the declaration of Helsinki.

### Population

A 3.5-year longitudinal study of TS was conducted in Kongwa district, Tanzania. The study has been previously described [12]. Women aged 15 and older were randomly selected from households in 48 communities within Kongwa based on a complete census of each village at baseline, in 2013. The participants were then contacted for repeat examination 3.5 years later, in 2016.

Kongwa district was chosen as it is a formerly trachoma hyperendemic district that recently achieved low TF prevalence as a result of concerted national and international efforts toward trachoma elimination. In 2008 Kongwa district had a prevalence of active trachoma (TF) of 31%; by 2013, the start of this current study, the overall baseline TF prevalence was 5.0% (95% CI 3.9, 6.1) and the TF prevalence remained at 5.0% at the end of the study. [13,14].

### Data collection

Data obtained on participants included a demographic survey followed by ocular photographs. Survey information collected on the participants included age and markers of socioeconomic status (SES) including level of formal education by head of household, bicycle ownership, presence of a latrine, cell phone ownership, presence of a mud roof and the time to walk to water as well as presence of an inside cooking fire. Interviewers were masked to the participant’s disease status.

#### TF assessment

The TF assessment has been described in greater detail previously [14]. Briefly, a trained grader performed an ocular examination of the right upper tarsal conjunctiva using a 2.5x loupe and flashlight to evaluate the presence of follicular trachoma in children ages 1–9 in the study communities at baseline. TF was graded as present or absent according to the WHO simplified grading scheme [15].

#### Scarring assessment

Photographs of the upper tarsal conjunctiva were obtained at baseline and follow-up. The photographs were graded for TS using a four-step severity scale based on a previously published grading scheme [16]. The scarring severity scale was defined as follows:

1. S1 (minimal), one or more lines of scarring at least 3 mm in length and some stellate scars, but total scarring occupying less than one eight of the upper eyelid (not as severe as S2).
2. S2 (mild), multiple lines or patches of scarring that occupy at least one eighth of the upper eyelid, but total scarring occupying less than one third of the upper eyelid (not as severe as S3).
3. S3 (moderate), scarring occupying at least one third of the upper lid with clear conjunctiva between, but total scarring occupying less than 90% of the upper eyelid (not as severe as S4).
4. S4 (severe), scarring occupying more than 90% of the conjunctiva.

The images were graded for scarring at 5x magnification. Images were assessed by two independent graders, and any disagreements that arose were openly adjudicated between the graders. A senior grader decided the final grade in cases where graders could not reach an agreement. Graders were masked to all other participant data and to each other’s grades prior to adjudication. Interobserver agreement in using this system of grading on a set of 60 images was unweighted kappa 0.65 between the two graders, and 0.70 and 0.67 between the two individual graders and the senior grader.

#### Scarring progression definition

Scarring progression was defined as any measurable worsening of existing scarring, thus an increase in scarring grade of at least one step or greater from baseline to follow up. Women with TS grades of S1, S2, or S3 at baseline were at risk of TS progression at follow-up.

#### Household poverty index

A household poverty index variable was created that was a summary score of the number of markers of low socioeconomic status that were present in a given household. Principle components analyses suggested that the first component was comprised of no formal education of the head of the household, lack of a bicycle, no cell phone, absence of a latrine, and presence of a mud roof; the second component included time to walk to water. We had a specific hypothesis that exposure to indoor cooking fire would be linked to increased risk of scarring progression, as such, this variable was left as an independent factor.

### Eligibility criteria

To be eligible for this study women had to be permanent residents of the community, have gradable upper tarsal conjunctival photographs, and be at risk for TS progression at baseline. Those with scarring grade of S4 at baseline were not eligible for progression as they had already reached maximum scarring severity.

### Statistical analysis

We estimated that with a fixed sample size of 646, calculated post-hoc, we had the ability to observe a TS progression of 35% with a precision of ± 4%.

Differences in age, markers of socioeconomic status (SES), presence of an inside cooking fire, and baseline scarring grade between participants and those lost to follow-up were analyzed using contingency tables. Significance was assessed using a Chi-squared and/or Fisher’s exact test.

Bivariate analyses compared age, markers of socioeconomic status, indoor cooking fire, and baseline TS severity between those with and without scarring progression. Exploratory analysis suggested that the relationship of age with TS progression was non-linear with a clear break at 50 years of age. Therefore, we used a spline regression with a node at age 50 to model the relationship of age with TS progression. Intraclass correlation coefficient (ICC) was used to evaluate village level clustering of TS progression.

Logistic regression models were created to evaluate potential predictors of scarring progression; the models created included an age-adjusted model and subsequent multivariable models. The initial multivariate model included age and age adjusted risk factors associated with TS progression with a p-value < 0.2. The final parsimonious multivariate model was created using a backward elimination approach. All analyses were conducted on SAS 9.4 software (SAS Institute, Cary, NC).

## Results

**Fig 1:**
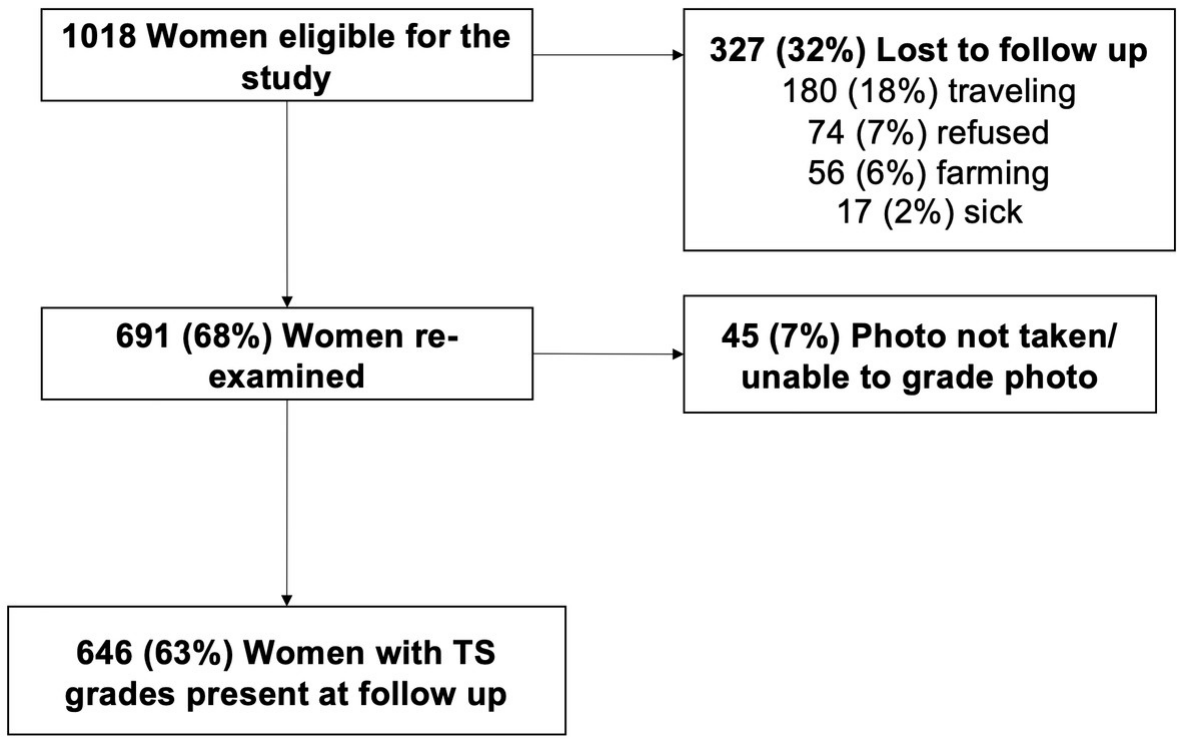
Participant follow-up.

At baseline, 1018 women aged 15 and older had TS grades at baseline and were at risk for TS progression. The median age of women was 41.7 years. 48.4% had S1, 36.9% had S2, and 14.6% had S3 scarring. Of the 1018 women, 691 (68%) were available for and completed follow-up assessments. The primary reasons for non-participation were travel for 180 (18%) individuals and refusal for 74 (7%) individuals. Forty-five women (7%) either could not have photos taken or their photos were ungradable (Fig 1). Participants and non-participants did not differ significantly in most of their baseline characteristics except that non-participants were less likely to have mild baseline scarring (p = 0.004) and more likely to have lived in a house with a mud roof (p = 0.003) (Table 1). No other baseline characteristics were significantly different between the two groups.

**Table 1:** Baseline characteristics by follow-up status.

Baseline characteristics	With follow-up grades N = 646*	No follow-up grades N = 372**	Overall N = 1018	p-value
Age in years, **mean (Standard Deviation (SD))**	41.6 (16.6)	41.9 (17.9)	41.7 (17.1)	0.82
Trachomatous Scarring severity grade, **n (%)**				
S1 (minimal)	290 (44.9)	203 (54.6)	493 (48.4)	**0.0004**
S2 (mild)	268 (41.5)	108 (29.0)	376 (36.9)	
S3 (moderate)	88 (13.6)	61 (16.4)	149 (14.6)	
Household characteristics, **n (%)**				
The head of the household has no formal education	312 (48.5)	202 (54.3)	514 (50.6)	0.07
No Latrine	139 (21.7)	89 (24.0)	228 (22.5)	0.39
No bicycle	377 (58.4)	220 (59.1)	597 (58.8)	0.85
Distance to water > 60 min	133 (20.6)	93 (25.1)	226 (22.3)	0.10
Mud roof	82 (12.7)	73 (19.6)	155 (15.3)	**0.003**
Inside cooking fire	186 (28.9)	116 (31.9)	302 (30.0)	0.33
No cell phone	410 (63.7)	226 (60.8)	636 (62.6)	0.36
Household poverty index, **mean (SD)**	2.05 (1.32)	2.17 (1.33)	210 (1.32)	0.15
Community Trachomatous Inflammation-Follicular prevalence in 1–9 year olds ≥5%, **n (%)**	318 (49.2)	178 (47.9)	496 (48.7)	0.67

* Missing values: 2 all household characteristics, additionally 2 latrine, 1 inside cooking fire, 2 household poverty index

**Missing values: 1 latrine,1 distance to water, 8 inside cooking fire, 1 household poverty index.

**Fig 2:**
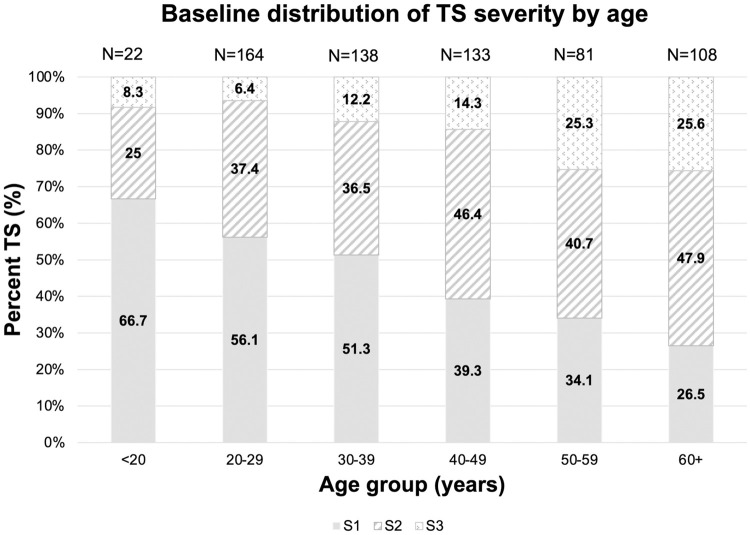
Baseline distribution of TS severity by age.

The cross-sectional severity of scarring by age was evaluated at baseline. 8% of participants less than 20 years of age had scarring of moderate severity (S3); the proportion of participants with moderate severity scarring increased to 26% in those 50 years of age and older (Fig 2).

**Fig 3:**
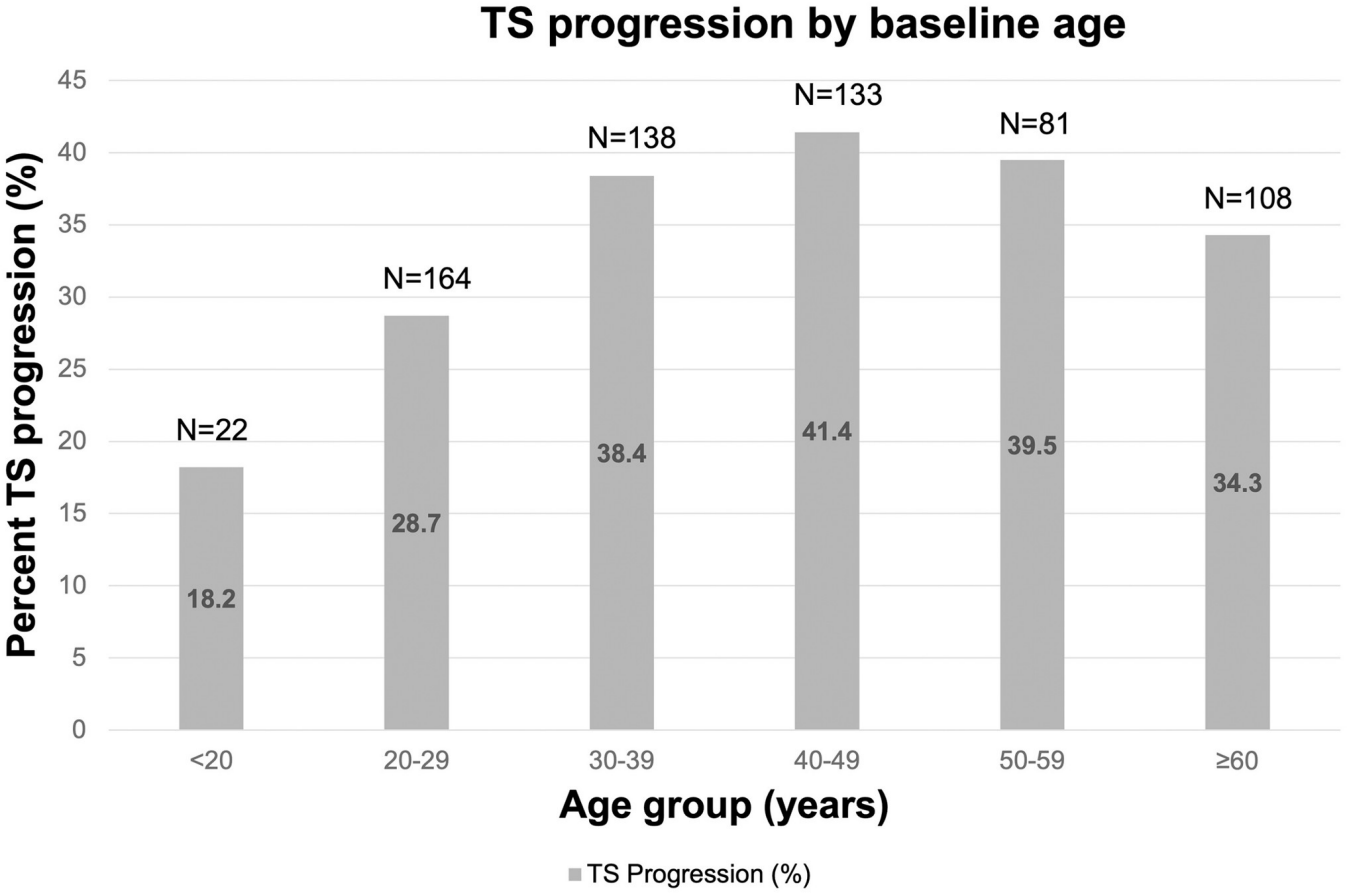
Cumulative progression of TS by baseline age.

The overall cumulative 3.5-year progression rate of scarring was 35.3% (95% CI 31.6–39.1) in this cohort, or approximately 10% per year. The cumulative progression rate of scarring increased steadily with age until age 50 (test for trend, p = 0.011) (Fig 3). In crude analyses, a statistically significant increased risk of progression of scarring was seen with four of the six markers of lower socioeconomic status including not owning a bicycle or cellphone, absence of a latrine, and presence of a mud roof (Table 2). The composite household poverty index showed a clear increased risk of TS progression with an increase in the number of markers that were present (test for trend, p<0.006). The presence of an indoor cooking fire also increased the risk of TS progression (p = 0.015). The baseline TF prevalence in the community where the women resided was not significantly associated with individual TS progression and there was no evidence of clustering of TS progression at the village level (ICC 0.05; 95%CI -0.01,0.11, p = 0.10).

**Table 2:** Baseline characteristics by Trachomatous Scarring (TS) progression.

Characteristic^+^	TS Progressed		p-value
	N	%	
Age in years			
<20	22	18.2	Spline, Age < 50, 0.011* Age > = 50, 0.28*
20–29	164	28.7	
30–39	138	38.4	
40–49	133	41.4	
50–59	81	39.5	
60 +	108	34.3	
Baseline TS severity			
S1 (minimal)	290	37.6	**0.01** *
S2 (mild)	268	38.4	
S3 (moderate)	88	18.2	
Education of Head of household			
No formal education	312	37.8	0.18
Some formal education	332	32.8	
Distance to water			
< 1 hour	511	33.9	0.15
1 hour or more	133	40.6	
Household bicycles			
None	377	38.7	**0.03**
At least one	267	30.3	
Latrine			
Absent	139	42.5	**0.04**
Present	503	33.2	
House roof			
Made of mud	82	51.2	**0.001**
Other materials	562	32.9	
Family owns at least one phone			
No	410	39.0	**0.008**
Yes	234	28.6	
Household poverty index**			
0–1	228	28.1	**0.006** *
2–3	323	37.2	
4+	91	46.2	
Cooking fire			
Outside/other building	457	32.4	**0.015**
Inside the house	186	42.5	
Trachomatous Inflammation-Follicular prevalence of 1–9 year olds in the community			
0%	61	27.9	0.59*
>0% to <5%	267	37.1	
≥5%	318	35.2	

^+^ Missing values: 2 all household characteristics, additionally 2 latrine, 1 inside cooking fire, 2 household poverty index

* Test for trend,

** Household poverty index = number of markers of low socioeconomic status for a household including: a lack of education for the head of household, no bicycle, no cellphone, no latrine, and a mud roof.

The age adjusted logistic regression predicting odds of TS progression, adjusted for baseline TS severity, showed a significant association of TS progression with older age in those younger than 50 years of age, an increased risk with an increase in household poverty index, and the presence of an indoor cooking fire (Table 3). The final parsimonious multivariate model, adjusting for baseline TS severity, included age and the household index. In those aged less than 50, for every year increase in age, the odds of TS progression increased by 3% (OR 1.03, 95% CI 1.01–1.05, p = 0.005). For every additional marker of low socioeconomic status in the household index, the odds of TS progression increased by 29% (OR 1.29, 95% CI 1.13–1.48, p = 0.0002).

**Table 3:** Potential risk factors associated with TS progression.

Characteristic	Age adjusted model of TS Progression* Odds Ratio (95% CI), p-value	Final multivariate model of TS Progression* Odds Ratio (95% CI), p-value
Age		
Women <50 (per year increase)	**1.03 (1.01, 1.04), 0.011**	**1.03 (1.01, 1.05), 0.005**
Women 50 or older (per year increase)	0.99 (0.96, 1.01), 0.28	0.98 (0.96, 1.00), 0.10
Household poverty index (per unit increase)	**1.28 (1.13, 1.46), 0.0003**	**1.29 (1.13, 1.48), 0.0002**
Cooking fire inside the house	**1.49 (1.04, 2.13), 0.03**	----------------
Distance to water>60min	1.33 (0.89, 1.97), 0.16	----------------

* Adjusted for baseline TS severity

## Discussion

In this cohort study of adult women, we found a cumulative scarring progression rate of 35.3% over 3.5 years in Kongwa, Tanzania, where the TF prevalence was 5%.

The current rate of scarring progression in Kongwa is very similar to what we observed a decade prior in a Kongwa village where the prevalence of TF was 29%; we found a scarring progression rate of 47.5% (95% CI 31.5, 63.9) over 5 years or approximately 9.5% per year [16]. Our current study found an annual scarring progression rate of approximately 10.1%. The lack of a difference in scarring progression rates despite TF prevalence dramatically dropping in this district suggests that the drivers of scarring progression are likely not related to on-going trachoma transmission in this district [3,10,17–20]. Indeed, others have also found no association of progression of scarring with presence of TF, but rather with inflammation or markers of inflammation [10,18,19].

Our study found that the rate of scarring progression increased significantly with age in women up to the age of 50. Previously published studies have shown that scarring incidence and prevalence increase with age [12,16]. Our study, however, is one of the first studies to find an association between scarring progression and age. The only other study to date that has found an association between scarring progression and age compared the mean age of individuals whose scarring progressed to those whose scarring did not progress in Tanzania and Ethiopia; the study did not look at age specific scarring progression rates [10]. The study had mixed results; in the Tanzanian cohort those whose scarring progressed were older, but in the Ethiopian cohort, there was no age difference [10].

Scarring progression may be associated with age due to biological factors that cause disease exacerbation in young and middle-aged women. Women are more likely to both develop more severe trachoma sequalae and to be disabled from these end-stage trachoma sequalae [5–7]. Women also have a higher prevalence of a variety of autoimmune conditions, which result from a disordered inflammatory immune response [21]. These autoimmune conditions often exacerbate during pregnancy and post-partum and ameliorate post-menopause [22]. If the inflammatory immune response that results after trachoma infection and leads to TS is viewed as disordered, then it is possible that there is a biologic corollary between auto-immune diseases and TS. If so, the relationship with age and TS that we are observing may be driven by biologic factors that result in women having a more severe inflammatory immune response in their reproductive years, which abates and leads to slower rates of TS progression as they become post-menopausal.

The association of scarring progression with age may also be a result of the S3 grade itself. Scarring progression is greater in the earlier stages of scarring; those with minimal (S1) and mild (S2) scarring at baseline have a higher rate of scarring progression than those with moderate (S3) scarring. The S3 category encompasses scarring involving from 1/3 up to 90% of the eyelid. Given the breadth of scarring encompassed in S3, it could take longer to progress through this severity level. As a result, individuals could be progressing within S3, but this would not be reflected in our progression data. Given that the prevalence of S3 increases until age 50, the leveling off in scarring progression seen in the older age groups could be a reflection of individuals progressing within S3 but not yet reaching S4.

Our study found that those with lower socioeconomic status (SES) had higher rates of scarring progression. To our knowledge, this study is the first to report an association between markers of SES and scarring progression. These findings are, however, consistent with other studies that have found an association between low SES and active trachoma, incident scarring, and/or trichiasis prevalence [12,23–29]. The relationship between low SES at baseline and higher rates of scarring progression is interesting and raises questions about possible mechanisms which could be contributing to this observation.

The association of low SES and scarring progression found in our study could reflect exposures participants had in childhood; this association could simply be a marker of the lower SES environment these women have lived in their entire lives. As active trachoma is more prevalent in lower SES households [23,24,26–30], participants who grew up in low SES households would have had more exposure to TF in childhood, leading to a greater likelihood of developing scarring and the inflammatory processes that drive scarring progression [10,31]. Childhood SES often determines future SES as an adult; this cycle of poverty is well described for low and middle income countries [32].

The association between low SES and scarring progression could also reflect risk from currently living in a low SES environment. Studies have shown an association of repeated conjunctival inflammation episodes with both incident and progressive scarring [10,31]. There are also studies that have found an association between low SES and low-grade systemic inflammation [33–35]. One study of children in the US found that low SES is associated with low-grade inflammation [33]. Another study of representative older American adults found an association between low SES and low-grade inflammation [34]. Both studies looked at C-reactive protein (CRP) as the marker of systemic inflammation, which is a non-specific marker; there were no studies that looked at more specific inflammatory markers, including those found in trachomatous scarring, and their relationship to SES. There have also been no studies to date evaluating ocular inflammation and SES. We posited that exposure to indoor cooking fire, known to cause ocular irritation and inflammation, would be linked to scarring progression, and it was in the crude and age- and TS grade- adjusted models. However, it was not a statistically significant predictor of progression when other markers of SES were included. The fact that the exposure to indoor cooking fire was not linked to progression in the multivariate model including the household poverty index may be because indoor cooking fire and the household poverty index are highly correlated; as such, we cannot draw a definitive conclusion as to whether or not indoor cooking fire plays a role in scarring progression.

However, exposures both in the present and in the past could be playing a role in the association between low SES and TS progression observed in this study. Interestingly, a study conducted on a cohort in Switzerland focusing on the relationship between a lifetime of SES inequity and adult systemic inflammation found that there was a cumulative effect of low SES over one’s lifetime with higher systemic inflammation levels in adults, determined by looking at CRP levels [35]. If in fact both childhood exposures and current exposures as indicated by SES markers are playing a role in the association observed between low SES at baseline and increased scarring progression, factors reflected by the markers of low SES would warrant further investigation. We did not evaluate ocular inflammation, which might have also enhanced the association with low SES.

A potential limitation is the loss to follow-up in our cohort. Non-participants were less likely to have mild baseline scarring and more likely to have lived in a house with a mud roof; both of those groups were more likely to have scarring progress, although there was no difference in any of the other markers of SES status. This may have resulted in our estimate of the overall rate of progression being conservative.

In conclusion, the 3.5-year progression of scarring among women in Kongwa, a formerly hyperendemic now turned hypoendemic district in central Tanzania was high at 35.3%; this scarring progression rate is despite a low active trachoma prevalence of less than 5%. This rate is very similar to the rate of scarring progression observed when the district was trachoma hyperendemic. Our findings suggest that once scarring has developed, it continues to progress even if trachoma rates in the district are low. Progressive scarring increased with lower SES and with age in those less than 50 years of age. Gaining a better understanding of all of the factors that are driving progression is important in determining if there are any factors that can be modified, and to help determine how long trichiasis surveillance and control programs will need to be continued to treat the burden of trichiatic disease, the downstream potentially blinding sequalae of scarring progression.

## Supporting information

S1 File STROBE checklist. (PDF)

S2 File Supporting data. (XLSX)
