# Supplementary material for: Risk factors for the progression of trachomatous scarring in a cohort of women in a trachoma low endemic district in Tanzania
Source: PLoS Negl Trop Dis. 2021 Nov 19;15(11):e0009914. doi: 10.1371/journal.pntd.0009914 (PMC8604323; doi:10.1371/journal.pntd.0009914)
Supplement: S1 File — (PDF) [file pntd.0009914.s001.pdf]

| Section/Topic | Item No. | Recommendation | Reported in Paper |
|---|---|---|---|
| **Title and abstract** | 1 | (*a* ) Indicate the study's design with a commonly used term in the title or the abstract | Page 2, Line 40 |
| | | (*b* ) Provide in the abstract an informative and balanced summary of what was done and what was found | Page 2, Line 23-57 |
| **Introduction** | | | |
| Background/rationale | 2 | Explain the scientific background and rationale for the investigation being reported | Page 4, Line 86-122 |
| Objectives | 3 | State specific objectives, including any prespecified hypotheses | Page 4, Line 124-126 |
| **Methods** | | | |
| Study design | 4 | Present key elements of study design early in the paper | Page 5, Line 133-152 |
| Setting | 5 | Describe the setting, locations, and relevant dates, including periods of recruitment, exposure, follow-up, and data collection | Page 5, Line 133-137 |
| Participants | 6 | (*a* ) Give the eligibility criteria, and the sources and methods of selection of participants. Describe methods of follow-up | Page 6, Line 199-202; Page 5, Line 134-137 |
| | | (*b* ) For matched studies, give matching criteria and number of exposed and unexposed | N/A |
| Variables | 7 | Clearly define all outcomes, exposures, predictors, potential confounders, and effect modifiers. Give diagnostic criteria, if applicable | Page 5-6, Line 161-195 |
| Data sources/ measurement | 8* | For each variable of interest, give sources of data and details of methods of assessment (measurement). Describe comparability of assessment methods if there is more than one group | Page 5-6, Line 147-195 |
| Bias | 9 | Describe any efforts to address potential sources of bias | Page 6, Line 178-179; Page 6, Line 220-221; Page 6, Line 223-228 |
| Study size | 10 | Explain how the study size was arrived at | Page 5, Line 136-137; Page 6, Line 199-202 |
| Quantitative variables | 11 | Explain how quantitative variables were handled in the analyses. If applicable, describe which groupings were chosen and why | Page 6, Line 210-228 |
| Statistical methods | 12 | (*a* ) Describe all statistical methods, including those used to control for confounding | Page 6, Line 207-228 |
| | | (*b* ) Describe any methods used to examine subgroups and interactions | N/A |
| | | (*c* ) Explain how missing data were addressed | N/A |
| | | (*d* ) If applicable, explain how loss to follow-up was addressed | Excluded from the study. Page 7, Line 248-251 |
| | | (*e* ) Describe any sensitivity analyses | N/A |
| **Results** | | | |
| Participants | 13* | (a) Report numbers of individuals at each stage of study—eg numbers potentially eligible, examined for eligibility, confirmed eligible, included in the study, completing follow-up, and analysed | Page 7, Line 246 |
| | | (b) Give reasons for non-participation at each stage | Page 7, Line 248-251 |
| | | (c) Consider use of a flow diagram | Figure 1 |
| Descriptive data | 14* | (a) Give characteristics of study participants (eg demographic, clinical, social) and information on exposures and potential confounders | Page 7 Line 247-248; Page 7 Line 251-255; Table 1 |
| | | (b) Indicate number of participants with missing data for each variable of interest | Page 8, Line 261-263, Table 2 |

| | | | |
|---|---|---|---|
| | | (c) Summarise follow-up time (eg, average and total amount) | Page 5, Line 136-137. |
| Outcome data | 15* | Report numbers of outcome events or summary measures over time | Page 7, Line 246-247; Page 8, Line 270-272, Figure 3 |
| Main results | 16 | (*a* ) Give unadjusted estimates and, if applicable, confounder-adjusted estimates and their precision (eg, 95% confidence interval). Make clear which confounders were adjusted for and why they were included | Page 11. Line 294-302, Table 3. |
| | | (*b* ) Report category boundaries when continuous variables were categorized | Page 5-6, Line 161-186 |
| | | (*c* ) If relevant, consider translating estimates of relative risk into absolute risk for a meaningful time period | N/A |
| Other analyses | 17 | Report other analyses done—eg analyses of subgroups and interactions, and sensitivity analyses | Page 11. Line 294-302, Table 3. |
| **Discussion** | | | |
| Key results | 18 | Summarise key results with reference to study objectives | Page 9, line 270-272 |
| Limitations | 19 | Discuss limitations of the study, taking into account sources of potential bias or imprecision. Discuss both direction and magnitude of any potential bias | Page 14, Line 402-406 |
| Interpretation | 20 | Give a cautious overall interpretation of results considering objectives, limitations, multiplicity of analyses, results from similar studies, and other relevant evidence | Page 12-14, Line 311-400 |
| Generalisability | 21 | Discuss the generalisability (external validity) of the study results | Page 12, Line 402-418 |
| **Other information** | | | |
| Funding | 22 | Give the source of funding and the role of the funders for the present study and, if applicable, for the original study on which the present article is based | In submission information |
